# Construction of IL-1 signalling pathway correlation model in lung adenocarcinoma and association with immune microenvironment prognosis and immunotherapy: Multi-data validation

**DOI:** 10.3389/fimmu.2023.1116789

**Published:** 2023-02-10

**Authors:** Ningning He, Yong Xi, Dongyue Yu, Chaoqun Yu, Weiyu Shen

**Affiliations:** ^1^ Department of Thoracic Surgery, Ningbo Medical Center Lihuili Hospital, Ningbo University, Ningbo, Zhejiang, China; ^2^ College of Life Sciences, Nankai University, Tianjin, China

**Keywords:** lung adenocarcinoma, tumour microenvironment, immunotherapy, single cell, immune checkpoint

## Abstract

Numerous studies have confirmed the inextricable link between inflammation and malignancy, which is also involved in developing lung adenocarcinoma, where IL-1 signalling is crucial. However, the predictive role of single gene biomarkers is insufficient, and more accurate prognostic models are needed. We downloaded data related to lung adenocarcinoma patients from the GDC, GEO, TISCH2 and TCGA databases for data analysis, model construction and differential gene expression analysis. The genes of IL-1 signalling-related factors were screened from published papers for subgroup typing and predictive correlation analysis. Five prognostic genes associated with IL-1 signalling were finally identified to construct prognostic prediction models. The K-M curves indicated that the prognostic models had significant predictive efficacy. Further immune infiltration scores showed that IL-1 signalling was mainly associated with enhanced immune cells, drug sensitivity of model genes was analysed using the GDSC database, and correlation of critical memories with cell subpopulation components was observed using single-cell analysis. In conclusion, we propose a predictive model based on IL-1 signalling-related factors, a non-invasive predictive approach for genomic characterisation, in predicting patients’ survival outcomes. The therapeutic response has shown satisfactory and effective performance. More interdisciplinary areas combining medicine and electronics will be explored in the future.

## 1 Introduction

The incidence and mortality of lung cancer remain high worldwide (1, 2). According to GLOBOCAN 2020 data, the incidence of lung cancer accounts for all cancers is 11.4%. The mortality rate is 18.0% (3), with non-small cell lung cancer (NSCLC) ranking first among malignant tumours in terms of incidence and mortality (4). The main pathological types include adenocarcinoma, squamous carcinoma and large cell carcinoma, which account for more than 85% of all lung cancers and have a 5-year survival rate of about 16% (5–7). Patients with advanced lung adenocarcinoma have a low survival rate (overall survival, OS) (8). Over- or under-treatment can significantly impact the overall outcome of lung cancer (9). Early diagnosis and treatment is the key to improving survival rates in lung cancer. According to the 8th edition of the tumour-node-metastasis (TNM) staging, patients with stage IA lung adenocarcinoma have a good prognosis after radical surgical resection, with an overall survival rate (OS) of over 90% at 5 years in stage IA1. However, the survival rate decreases with increasing T-stage, with a 5-year OS of 77% in stage IA3 (10). Previous studies have shown that there is no survival benefit from adjuvant chemotherapy (ACT) in stage IA patients (11), so ACT in stage IA is not recommended by current guidelines (12, 13). Therefore, the treatment outcome for lung cancer is not ideal. With the in-depth research on the molecular biology and genetic level of tumours, the development and application of targeted drugs have driven the progress of NSCLC treatment and significantly improved the prognosis of patients (14). Therefore, early diagnosis and treatment are crucial for patients with lung adenocarcinoma.

Inflammation is known as the “seventh hallmark of cancer” (15–17), and many studies have shown that inflammation plays an essential role in the development and progression of cancer (18, 19), among which IL-1 is one of the classical inflammatory factors. Plays a vital role in the development of cancer (20). However, relatively few studies use IL-1 as a prognostic marker in lung adenocarcinoma.

In this study, we aimed to develop a transcriptomics-based approach to calculate immune cell scores and predict survival outcomes in lung adenocarcinoma patients. We collected lung adenocarcinoma transcriptional profiling data and corresponding clinical information from the GDC, TISCH2 and GEO databases, explored the level of immune cell activation in lung adenocarcinoma by obtaining differential genes at the expression level, constructed a prognostic model for lung adenocarcinoma based on IL-1-associated genes, and identified several IL-1-associated key genes as potential biomarkers. Our findings reveal a crucial role for IL-1 in lung adenocarcinoma, and we propose a convenient method to help diagnose and predict survival outcomes of lung adenocarcinoma patients.

## 2 Materials and methods

### 2.1 Data download and pre-processing

Obtain the FPKM expression matrix for lung adenocarcinoma from the Genomic Data Commons (GDC) repository (https://portal.gdc.cancer.gov/ ). Gene expression profiles of lung adenocarcinoma were queried from the GEO Gene Expression Dataset (https://www.ncbi.nlm.nih.gov/geo/ ) and microarray and high-throughput sequencing transcriptome data GSE42127, GSE72094, GSE26939, GSE31547, GSE19188, GSE14814, GSE37745, GSE5828 (BULK transcriptome data for validation), GSE135222, PRJEB23709, phs000452 (BULK transcriptome data for validation of impact on immunotherapy); we applied a text-mining-based data parsing workflow to collect the TISCH2 database (http://tisch.compgenomics.org/ ) of GSE117570 lung adenocarcinoma single-cell dataset, with all genes expressed in at least 3 cells, at least 200 genes per cell, UMIs retaining reads in the range 500-6500 depending on distribution, and percentage of mitochondrial reads < 80%; single-cell data as UMI transcript matrices as well as cellular information matrices, merged into Seraut objects required for analysis, using LogNormalize to normalise the data and check for batch effects between samples by UMAP and found no significant batch effects between samples.

### 2.2 IL-1 molecular typing and signature construction for lung adenocarcinoma

The IL-1 gene set was sourced from an exhaustive literature review (**Table S1**), and a conserved molecular typing of lung adenocarcinoma was constructed using non-negative matrix factorization (NMF) to identify the optimal number of subgroups based on the number of clusters from 2-10. To assess the correlation between TCGA-LUAD prognosis and subgroups, the expression of IL-1 signalling genes in LUAD samples was demonstrated by heat map in order to further resolve the respective IL-1 molecular profiles within different subgroups.

### 2.3 Differential expression and enrichment analysis

Based on the conserved typing of IL-1 signalling, GSVA was performed on samples in TCGA-LUAD to calculate cancer hallmarks scores and assess cancer hallmarks scores between groups. Feature construction was based on differentially expressed genes identified in the IL-1 gene set in the best prognosis group versus the worst prognosis group. FDR-corrected p-values adj. p < 0.05 and log2FC absolute > 0.5 were used as thresholds to select IL-1 genes essential prognostic genes, and the prevalent cancer suppressor function was identified for high scoring status in the independent validation set using IL-1score and cancer hallmarks scores from eight independent validation datasets for correlation.

### 2.4 Validation of prognostic potency of IL-1 in the lung cancer dataset

The predictive power of IL-1score in lung adenocarcinoma patients was assessed in 510 patients from TCGA-LUAD and eight additional lung cancer datasets collected separately using the GSVA algorithm to calculate sample IL-1score based on median grouping.

### 2.5 Immune infiltration

SSGSEA was used to assess the abundance of 28 immune cells in TCGA-LUAD samples as well as in tumour samples from the independent validation set, and gene expression matrix data (TPM) was uploaded to CIBERSORTx for further CIBERSORTx immune infiltration analysis using the R package IBOR (0.99.9) based on MCPcounter, xCell, and EPIC immune cell infiltration algorithms to calculate immune infiltration scores, generate immune cell infiltration matrices, calculate spearman correlations of immune function, immune cells and immunotherapy efficacy indicators with IL-1score, and assess the predictive effect of IL-1score in immunotherapy.

### 2.6 Drug sensitivity analysis

The Genomics Database of Anticancer Drug Sensitivity (GDSC) data collection The GDSC (https://www.cancerrxgene.org/ ) is a public database for studying drug sensitivity and drug response targets in cancer cells. The GDSC currently contains data on the response of more than 1000 cell lines to more than 200 drugs (21). Based on the GDSC database, the expression data of lung adenocarcinoma cell lines were extracted, and IL-1score was calculated for each cell line and grouped based on median; the AUC and IC50 data of multiple drugs in cell lines were combined, and their correlation with CAF related signature was calculated using Spearman correlation.

### 2.7 Differential expression and enrichment analysis

Expression profiling microarray analysis was performed using the Illumina NextSeq assay platform based on the GSE117570 lung adenocarcinoma single-cell dataset. Raw datasets were used before the Rupee R package to ensure quality control (QC) results. The number of genes detected within each cell (nFeature RNA) and the total number of molecules detected within the cell (nCount RNA) were calculated. These two gene counts were compared to the number of mentions procured by sequencing within each cell. We assessed the widespread mitochondrial genomic contamination in low-quality or dead cells by calculating reads paired with the mitochondrial genome using a percentage feature set function. Cells were clustered using Unified Flow Interpolation and Projection (UMAP) downscaling techniques, each cell subpopulation was visualised against other cells using FindAllMarkers, and the distribution of IL1-score in single-cell downscaled space was calculated using GSVA. Subsequently, cell subpopulations were divided by median IL-1score, primary aggregated cells in high-scoring subpopulations were observed, and GSEA pathway enrichment analysis was performed using the R package cluster profile (4.2.2).

### 2.8 Immunohistochemistry

Tissue sections from eight lung cancer patients were selected for immunohistochemistry for labeling the proteins IL-1β and IL-18. specimens were dewaxed in xylene solution and graded alcohol. The sections were placed on a sectioning rack and soaked in three xylene solutions for 20 min, then in solutions of 100% alcohol, 95% alcohol, 85% alcohol and 75% alcohol for 5 min, respectively. ddH2O was washed three times for 3 min each time. for antigen repair, water was heated in an induction cooker to boiling, then in 0.01 M sodium citrate buffer (pH 6.0) for 5 min The sections were slowly placed in 0.01M sodium citrate buffer (pH 6.0) and heated to the top of the autoclave valve for 3min. after which they were cooled naturally at room temperature for about 30min. The sections were removed at room temperature and washed three times with PBS buffer for 5 min each. 3% H2O2 was used to cover the tissue specimens on the slides and incubated at room temperature for 20 min to block the effect of endogenous peroxidase on the experiments. 3 times with PBS buffer for 5 min each. 10% H2O2 was used to cover the tissue specimens on the slides and incubated for 20 min at room temperature to block the effect of endogenous peroxidase on the experiments. The tissue specimens were incubated for 40-60 min at room temperature in a wet incubation chamber. 10% goat serum blocking solution was used to prepare recombinant Anti-IL-1 beta antibody (abcam, UK, ab283818) at a ratio of 1:500 and recombinant Anti-IL-18 antibody (abcam, UK, ab243091) at a ratio of 1:1000, respectively. ab243091), and the appropriate amount of antibody was added dropwise to the tissue specimen, arranged in an incubation wet box, and placed in a 4°C refrigerator for overnight incubation. The next morning, the specimens were removed, washed three times with PBST buffer for 5 min each time, and then rinsed with PBS buffer for 5 min. Horseradish peroxidase-labeled goat anti-rabbit antibody was prepared in 10% goat serum blocking solution at a ratio of 1:50 (Biyuntian Biotechnology, China, Cat No. A0208), and then an appropriate amount of antibody was added dropwise to the tissue specimens and incubated at room temperature for 1 h. PBST buffer Wash 3 times for 5 min each time, then wash again with PBS buffer for 5 min. Add solution A and solution B of DAB Horseradish Peroxidase Color Development Kit (China Biotec, Cat No. P0202) to the light-proof EP tube at a volume ratio of 1:1 and mix well. The sections were dehydrated in 75% alcohol, 85% alcohol, 95% alcohol and 100% alcohol for 5 min each, then soaked in three xylene solutions for 10 min, and sealed with neutral gum. The specimen sections were observed under the microscope for staining, scored according to the scoring method, and the results were recorded and photographed for preservation.

### 2.9 Statistical and survival analysis

In the TCGA database and GEO validation dataset, groupings were made based on the best cutoff of score or gene expression calculated by the survminer. The impact on prognosis was assessed by constructing Kaplan-Meier curves and logrank tests using the survival R package. All statistical tests for significance were p < 0.05.

## 3 Results

### 3.1 Construction and survival validation of IL-1 typing in lung adenocarcinoma

**Figure 1:**
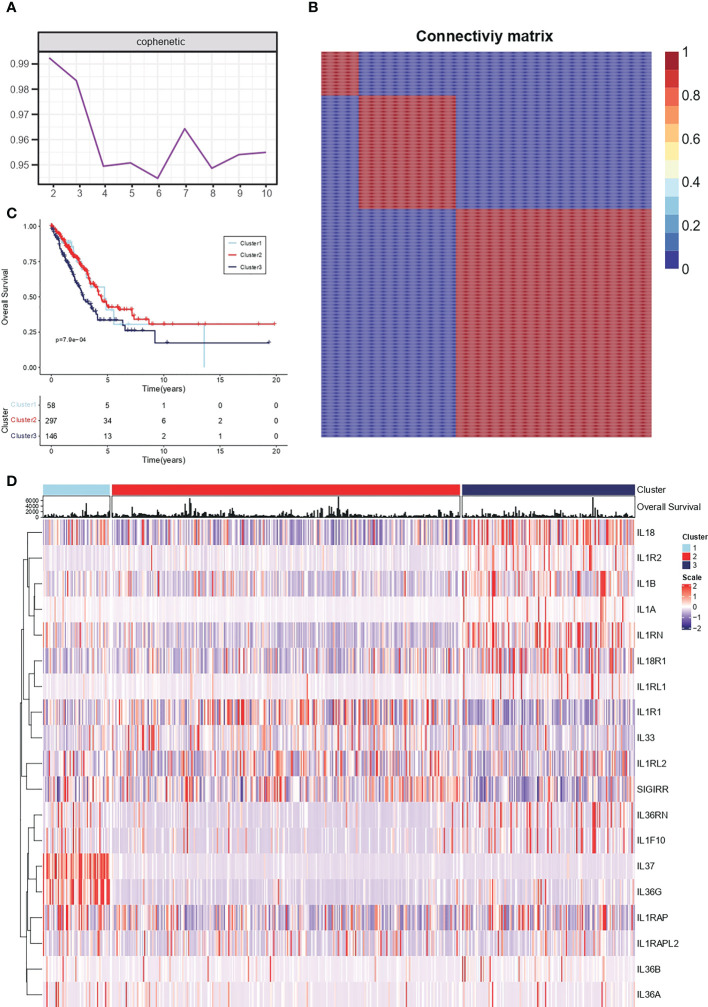
Conserved molecular typing of IL-1 signalling in lung adenocarcinoma. **(A)** Factorization rank for 2-10 clusters. **(B)** Heatmap of the gene expression of three clusters. **(C)** K-M curves of different IL-1 subtypes of lung adenocarcinoma. **(D)** Molecular characteristics of IL-1 among different clusters of TCGA-LUAD.

Conservative IL-1 subtype differentiation was performed by non-negative matrix decomposition. The best rank = 3 was determined by the previous point of the decreasing maximum co-relation coefficient (**Figure 1A**). 510 individual lung adenocarcinoma samples in TCGA-LUAD were divided into three conserved subtypes (**Figure 1B**). Survival analysis revealed that the grouping based on IL-1 signalling-related genes had a prognostic differentiation, with group 2 having the best prognosis and group 3 the worst (**Figure 1C**). To further resolve the respective IL-1 molecular profiles within the different subgroups, the expression of IL-1 signalling genes in LUAD samples was demonstrated by heat map (**Figure 1D**).

### 3.2 Functional resolution of IL-1 molecular typing and IL-1score construction

**Figure 2:**
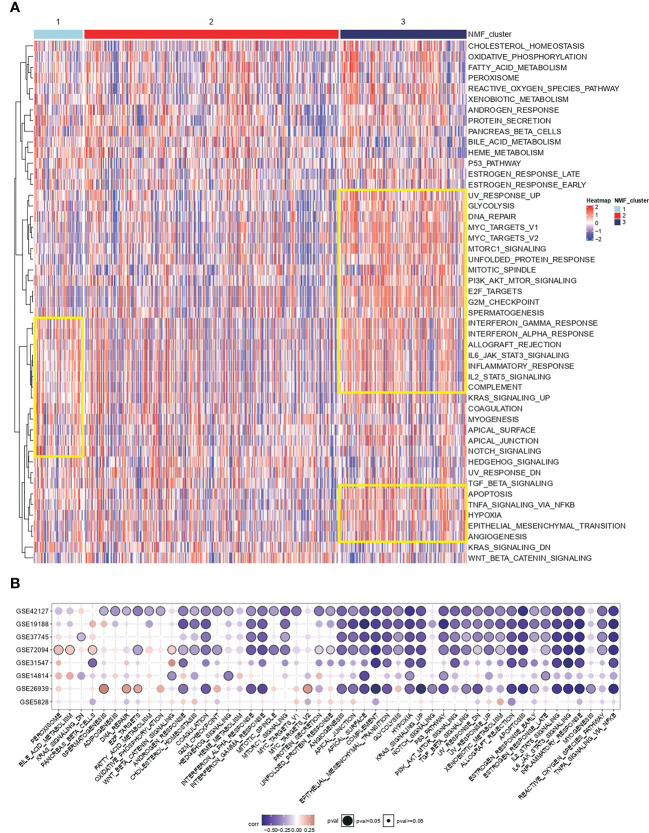
Functional resolution of IL-1 molecular typing and IL-1score construction. **(A)** The respective cancer hallmarks pathway scores among different subgroups of TCGA-LUAD. **(B)** Correlation of IL-1score with cancer hallmarks pathway scores from eight independent validation datasets.

Cancer hallmark is an oncogenic pathway that accumulates gradually during cancer development. For the conservative typing of IL-1 signalling in **Figure 1**, GSVA was performed on samples in TCGA-LUAD to calculate cancer hallmark scores. It was seen that cancer hallmarks pathway scores were higher in both types with poorer prognosis (group 1 vs group 3) and lower in group 2 (**Figure 2A**). Based on the differentially expressed genes in the IL-1 gene set in the best prognosis group (group 2) versus the worst prognosis group (group 3) to determine the characteristic gene set for group 3, FDR-corrected p-values adj. p < 0.05 and log2FC absolute values > 0.5 were selected as thresholds, and the final genes selected were IL-1B, IL18, IL1RN, IL1R2 and L1RAP. Using the GSVA algorithm, The scores of the samples in TCGA-LUAD based on the expression of the above genes were calculated and named IL-1score. The prevalent cancer suppression function was determined for high scoring status in the independent validation set using IL-1score and cancer hallmarks scores from the eight independent validation datasets for correlation (**Figure 2B**).

### 3.3 Validation of prognostic potency of IL-1 in lung cancer dataset

**Figure 3:**
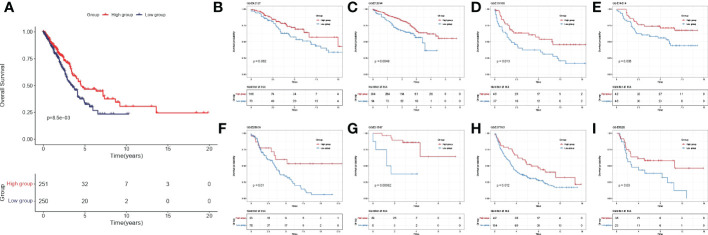
Validation of prognostic efficacy of IL-1 in the lung cancer dataset. **(A)** K-M curves of the TCGA-LUAD cohort based on IL-1score **(B-I)** K-M curves of the eight independent validation datasets based on IL-1score.

Among 510 lung adenocarcinoma patients in TCGA-LUAD, IL-1score could differentiate prognosis based on median grouping. The population in the higher IL-1score group was found to generally have a longer prognosis and better survival (**Figure 3A**). Meanwhile, in eight additional lung cancer datasets collected separately using the GSVA algorithm to calculate the sample IL-1score, the higher IL-1score group had a better prognosis than the lower group, the same as the TCGA-LUAD results (**Figures 3B-I**).

### 3.4 Dissecting the immune profile of IL-1 signalling

**Figure 4:**
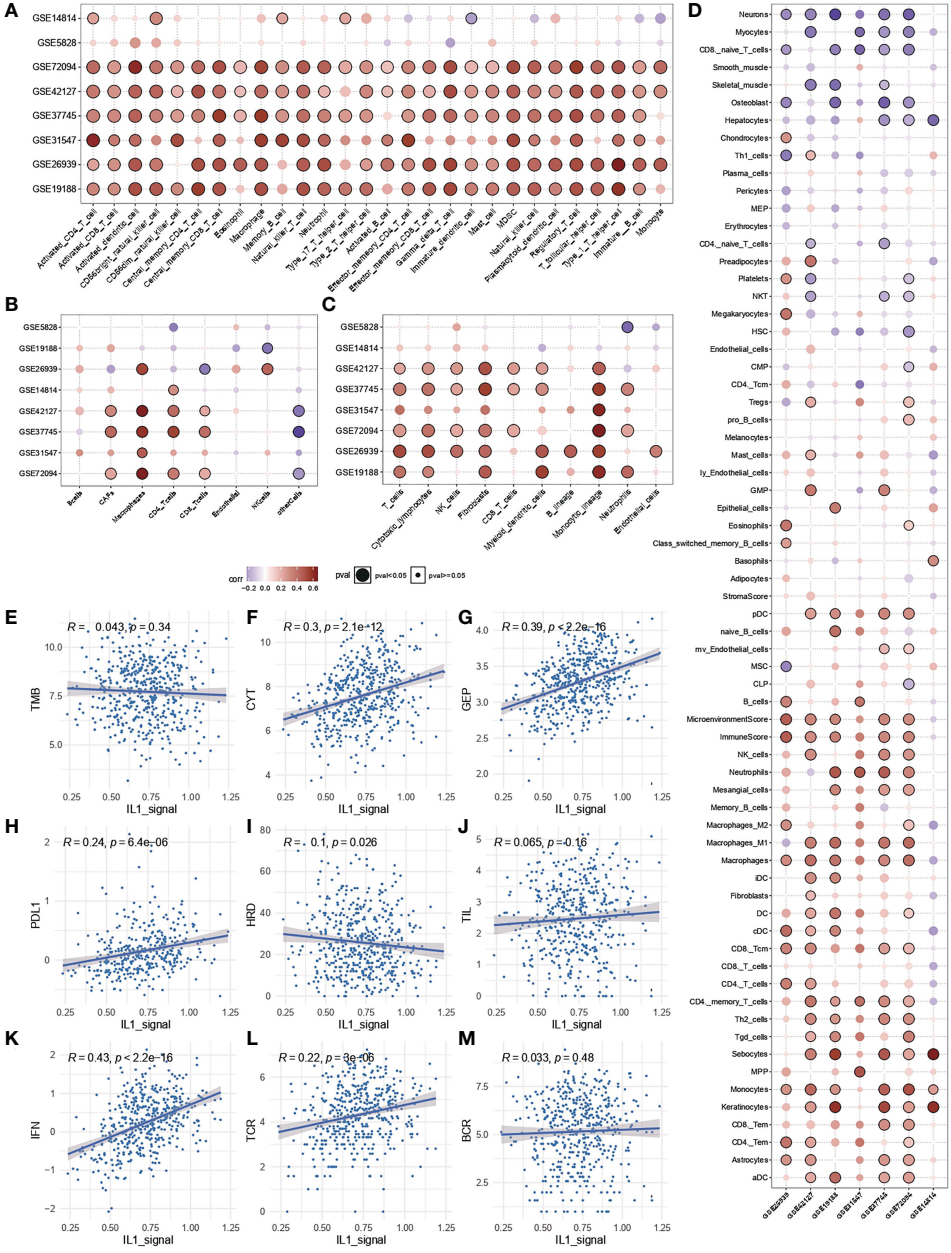
Immunological characterisation of IL-1 signalling. **(A-C)** Correlation of IL-1score with immune function activation in multiple datasets. **(D)** Correlation of IL-1score with immune cell activation. **(E-M)** Correlation of IL-score with multiple pan-immune indicators.

To further clarify the source of the prognostic, predictive power of IL-1 signalling, enrichment analysis of immune infiltration and calculation of spearman correlation of immune cell enrichment with IL-1score were performed for TCGA-LUAD samples as well as for the independent validation set. The results showed that the IL-1score exhibited a general immune activation efficacy across multiple datasets in various immune infiltration algorithms based on ranking and deconvolution algorithms, as evidenced by a broad positive correlation with immune cell infiltration (**Figures 4A-C**). Further breakdown of cellular components identified a general positive correlation of IL-1 in effector and activating immune cells (e.g. Th1, CD4T, CD8T) and a negative correlation in suppressive (naive) immune cells (e.g. monocytes, CD8Tnaive) (**Figure 4D**). The IL-score was positively correlated with several pan-immune markers, particularly in several immunotherapy efficacy markers (GEP, CYT, PDL1), immune infiltration (TIL), interferon signalling (IFN), TCR and BCR. No correlation was observed between tumour mutational load (TMB) and homologous recombination deficiency (HRD) (**Figures 4E-M**), suggesting that IL-1 signalling acts mainly by enhancing the anti-tumour function of immune cells and has little effect on the tumour cells themselves.

### 3.5 Predictive effects of IL-1score in immunotherapy

**Figure 5:**
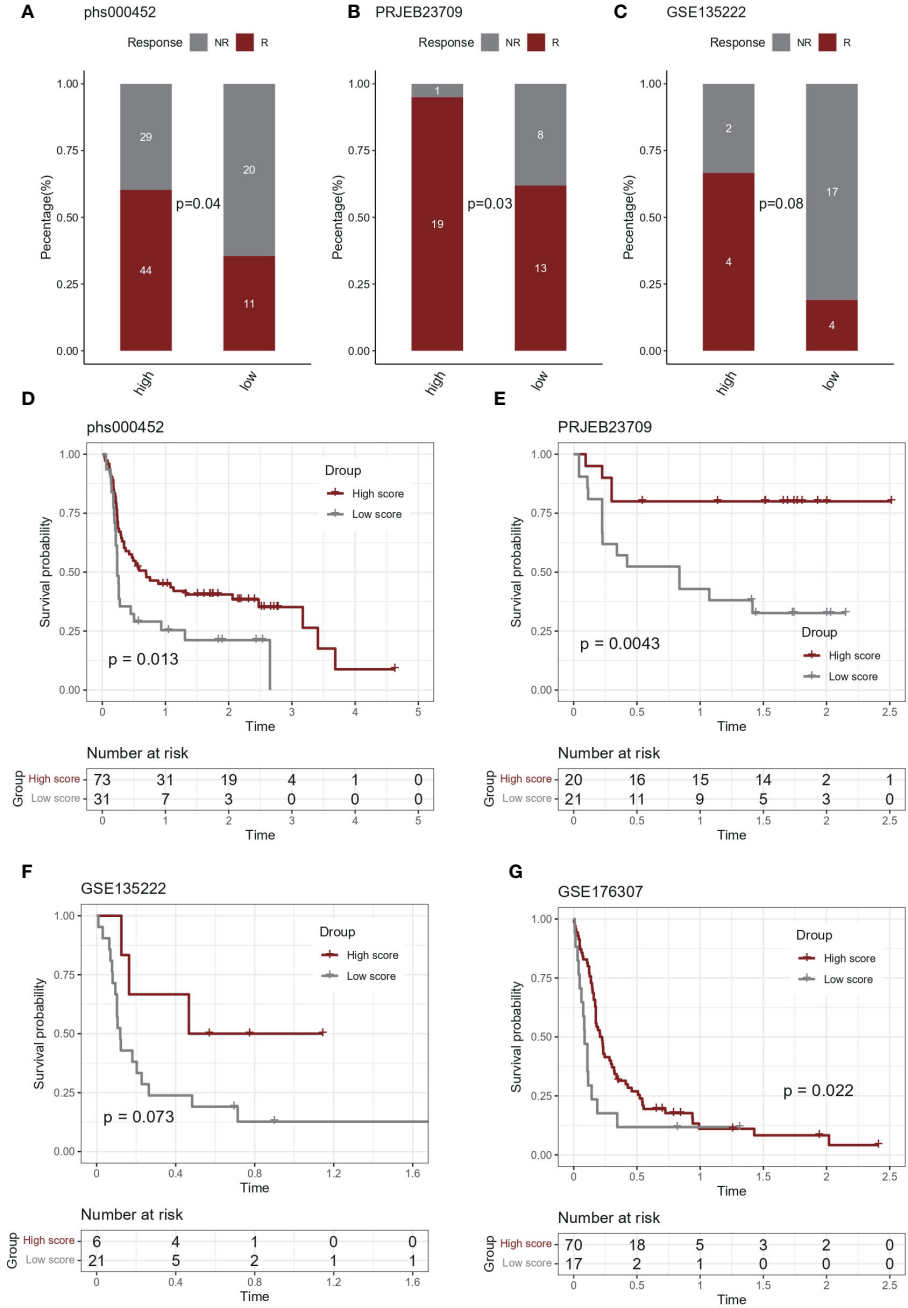
Predictive effect of IL-1score in immunotherapy. **(A-C)** In the immunotherapy cohort, IL-1score showed discriminatory ability for immunotherapy efficacy. **(D-G)** In terms of survival, the group with high IL-1score showed an advantage in progression-free survival.

In the immunotherapy cohort, IL-1 score showed discriminatory ability for immunotherapy efficacy (chi-square test, **Figures 5A-C**). In terms of survival, after grouping based on IL-1score, the group with a high IL-1score demonstrated an advantage in progression-free survival (**Figures 5D-G**).

### 3.6 IL-1score-based drug sensitivity speculation

**Figure 6:**
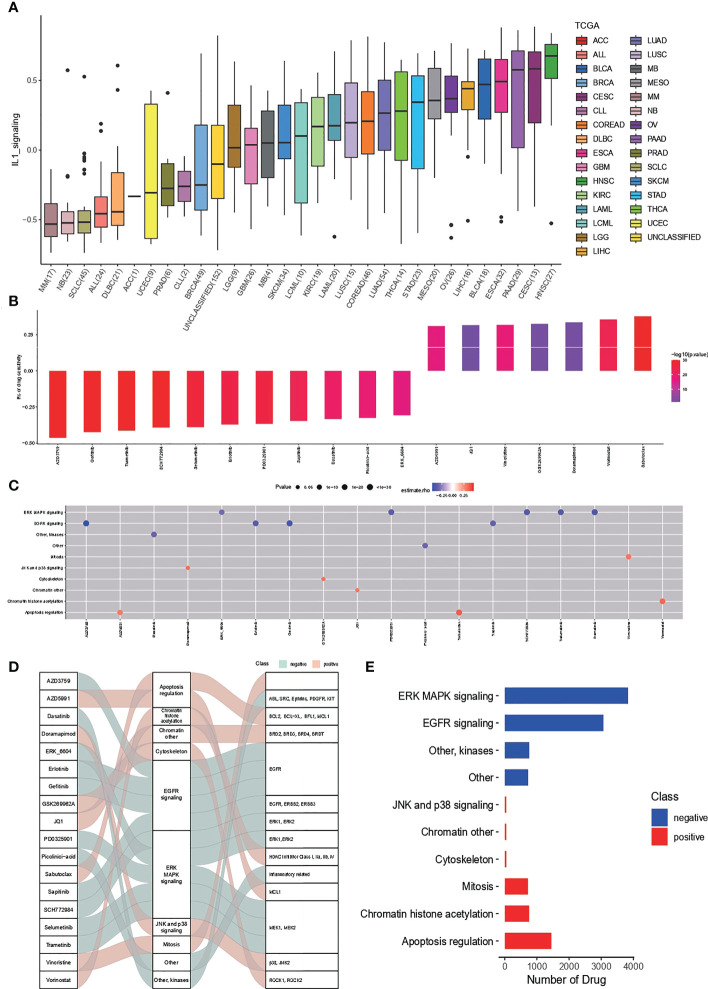
Drug sensitivity of IL-1score. **(A)** Distribution of IL-1score among different cancer cell lines in the GDSC cancer cell line database. **(B, C)** Drug sensitivity analysis screened for multiple drugs positively correlated with IL-1score scores. **(D)** Sankey diagrams of drugs with corresponding targets and pathways of action. **(E)** Major synergistic pathways between IL-1score and drugs.

Drug sensitivity analysis based on IL-1score speculation is shown in **Figure 6**. The distribution of IL-1score among different cancer cell lines in the GDSC cancer cell line database was first observed (**Figure 6A**). Head and neck squamous carcinoma had the highest score, with LUAD at a median of 11 out of 33 cancers. Further drug sensitivity analysis screened for multiple drugs positively correlated with IL-1score scores, suggesting the possibility of drug conjugation (**Figures 6B, C**). The Sankey diagrams of drugs with corresponding targets and pathways of action are shown in **Figure 6D**. The synergistic effects of IL-1score with drugs are mainly seen in drugs that regulate the apoptotic process, histone acetylation, and drugs that act on the mitotic process. In contrast, drugs that act on ERK-MAPK signalling and EGFR signalling should not be used in patients with high IL-1score, and their efficacy may be less than expected (**Figure 6E**).

### 3.7 Single-cell transcriptome resolution of IL-1 signalling in lung adenocarcinoma

**Figure 7:**
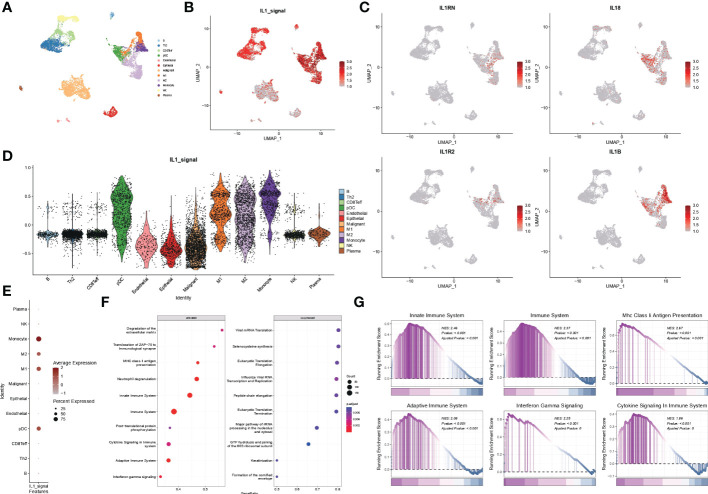
scRNA-seq data from GSE117570. **(A)** Single-cell analysis of UMAP from lung adenocarcinoma patients divided into 12 cell subgroups. **(B)** Distribution of IL1-score in single-cell descending space. **(C)** Specific expression of individual subtypes of IL-1 in various types of immune cells. **(D)** Violin diagram showing the distribution of IL-1score in various subpopulations of cells. **(E)** Dot plots showing the percentage expression of immune cell subpopulations. **(F, G)** Functional analysis of myeloid cells using GSEA.

**Figure 8:**
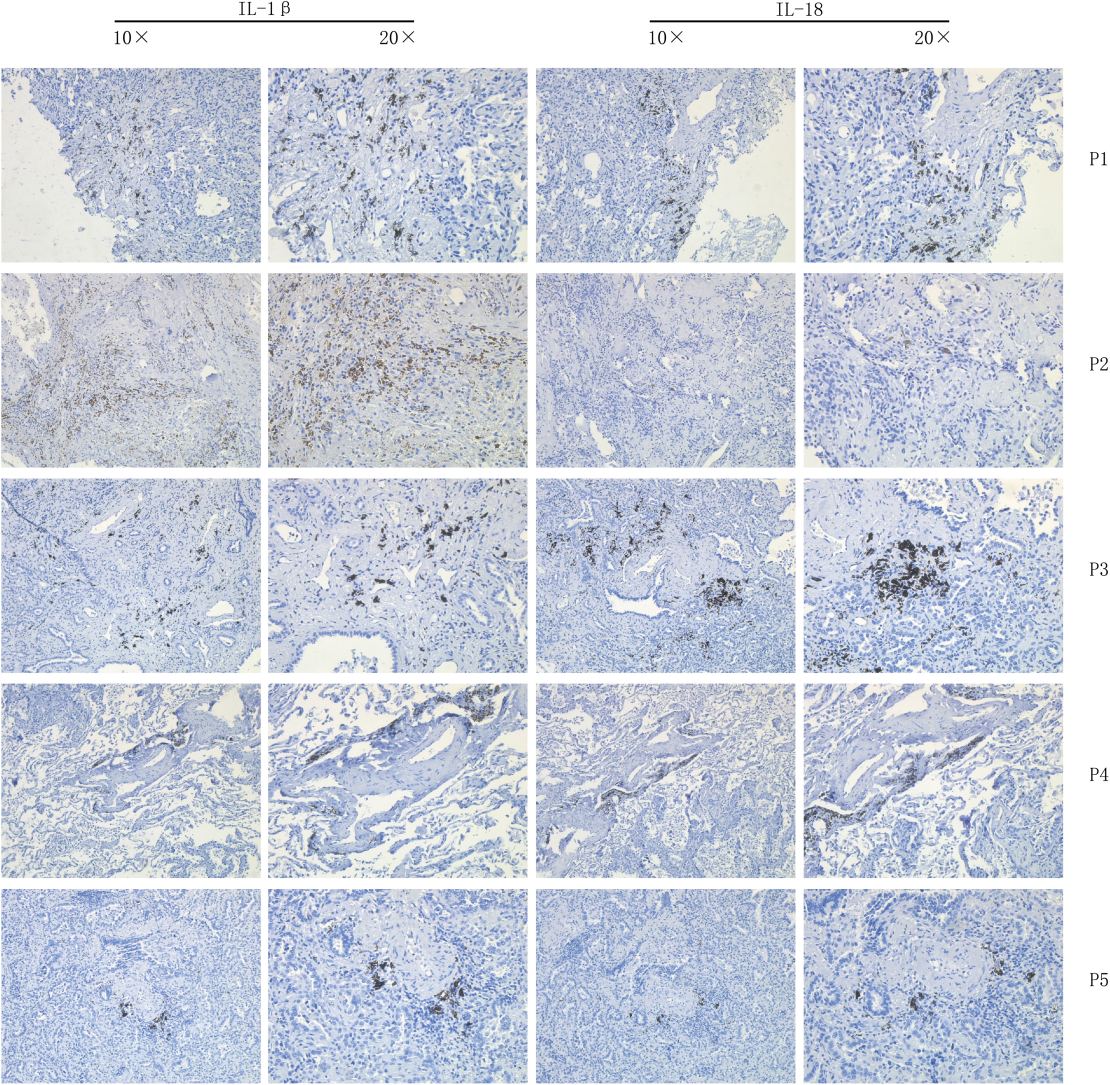
Immunohistochemistry.

Malignant, stromal and immune cells within various tumour microenvironments such as B cells, Th2 cells, CD8T cells, pDC cells, endothelial cells, epithelial cells, malignant cells, M1 macrophages, M2 macrophages, monocytes, NK cells, and plasma were annotated using UMAP downscaled as in **Figure 7A**. The distribution of the IL1 score in single-cell descending space was calculated using GSVA and found to be predominantly distributed in immune cells (**Figure 7B**). Resolution for the fraction also demonstrated specific expression of IL-1B and IL-18 in immune cells, particularly myeloid cells (**Figure 7C**). The distribution of IL-1score in each subpopulation was observed using violin plots, which showed a generally high expression in myeloid cells (**Figure 7D**). In contrast, the percentage of expression in the M1 subpopulation was higher than in the M2 subpopulation in the dot plots, suggesting a function of promoting M1 directional polarisation and thus enhancing anti-tumour immunity (**Figure 7E**). Subsequently, to verify the function of IL-1 signalling at the single cell level, cell subpopulations were classified by median IL-1score, with the high-scoring subpopulation being mainly myeloid cells. Myeloid cells were extracted and analysed for function using GSEA, demonstrating the enrichment pathways in the top 10 and bottom ten enrichment scores (**Figure 7F**). The functions were mainly enriched in the extracellular matrix, antigen immune presentation, activation of the immune system (innate/acquired), cytokine secretion, and interferon signalling (**Figure 7G**), suggesting a function of IL-1 signalling to promote antigen presentation and thus enhance tumour immunity, consistent with the bulk transcriptome results (**Figure 8**).

## 4 Discussion

Lung adenocarcinoma is a tumour type that poses a severe threat to human health. Most of its early symptoms are not obvious, and most are already in the middle to late stages when detected. The 5-year survival rate remains low (22, 23). Therefore, identifying potential biomarkers and elucidating the molecular mechanisms underlying their development can help in the early diagnosis of tumours and improve their prognosis (24–26). Available bioinformatics analyses can be a powerful tool for identifying biomarkers and therapeutic targets related to tumour progression and treatment. Previous studies have shown that novel serum biomarkers, including serum exosomes (27–29) and serum CEA (30–33), are good predictors of prognosis in lung adenocarcinoma. In addition, serum biomarkers related to inflammatory response are also good predictors of lung adenocarcinoma prognosis (34, 35). IL-1, one of the inflammation-related factors, has not been reported as a prognostic marker for lung adenocarcinoma.

We downloaded TCGA-LUAD samples from the GDC, GEO, TISCH2 and TCGA databases and eight independent validation sets of data related to lung adenocarcinoma patients. We classified 510 individual lung adenocarcinoma samples from TCGA-LUAD into three conserved subtypes based on the IL-1 gene set from an exhaustive literature review source, with conservative IL-1 subtype differentiation by non-negative matrix decomposition. We further calculated the cancer hallmark pathway scores within the different subgroups. We showed that the cancer hallmark pathway scores were higher in the two types with poorer prognoses (group 1 and group 3) and lower in group 2. We identified the characteristic gene set for group 3 based on the differentially expressed genes in the IL-1 gene set in the best prognosis group (group 2) and the worst prognosis group (group 3). The final selected IL-1-related essential gene genes were IL-1B, IL18, IL1RN, IL1R2 and L1RAP, and the GSVA algorithm was used to calculate the expression of the above genes based on the samples in TCGA-LUAD. The prediction model was constructed using the GSVA algorithm to calculate the scores of samples in TCGA-LUAD based on the expression of the above genes, named IL-1score.

We further calculated the IL-1score of samples in TCGA-LUAD and eight lung cancer datasets using the GSVA algorithm, distinguishing between high and low groups, observed the correlation between IL-1score and the prognostic amount and found that the population in the higher IL-1score group generally had a longer prognosis and better survival. To further clarify the correlation between IL-1 signalling and immune We performed an immune infiltration correlation analysis, which showed that among multiple immune infiltration algorithms based on rank-ranking and deconvolution algorithms, IL-1score presented a general immune activation efficacy across multiple datasets, reflected in a broad positive correlation to immune cell infiltration. Further breakdown of cellular components identified a general positive correlation between IL-1 in effector and activating immune cells, such as Th1, CD4T, and CD8T, but a negative correlation with suppressive immune cells, such as monocytes, CD8Tnaive, and based on this, further resolution of pan-immune indicators, the IL-score was positively correlated with several pan-immune indicators. This was particularly evident in several immunotherapy efficacy indicators (GEP, CYT, PDL1), immune cell infiltration (TIL), interferon signalling (IFN), TCR and BCR. No correlation was observed between TMB and Homologous Recombination Deficiency, suggesting that IL-1 signalling mainly enhances immune cells’ anti-tumour function and has little effect on the tumour cells themselves. In the immunotherapy cohort, IL-1score presented a discriminatory ability for immunotherapy efficacy. In terms of survival, after grouping based on IL-1score, the group with a high IL-1score showed an advantage in progression-free survival.

Drug sensitivity analysis based on IL-1score showed that the synergistic effect of IL-1score with drugs was mainly seen in drugs regulating the apoptotic process, histone acetylation, and drugs acting on the mitotic process, while drugs acting on ERK-MAPK signalling and EGFR signalling should not be used in patients with high IL-1score. Their efficacy may be less than expected. The results of the single-cell transcriptome analysis showed that IL1-score is mainly distributed in immune cells in the single-cell descending space. The analysis of the fractional components also demonstrated the specific expression of IL-1B and IL-18 in immune cells, especially in myeloid cells, with a higher percentage of expression in the M1 subpopulation than in the M2 subpopulation in the dot plot, suggesting that it promotes M1 directional polarisation and thus enhances anti-tumour immunity. Myeloid cells were extracted and analysed for function using GSEA, which revealed that their functions were mainly enriched in the extracellular matrix, antigen immune presentation, activation of the immune system (innate/acquired), cytokine secretion, interferon signalling and other functions, suggesting that IL-1 signalling promotes antigen presentation and thus enhances tumour immunity, in agreement with the bulk transcriptome results.

## 5 Conclusion

In conclusion, this study investigated the predictive role of IL-1 signalling-related genes on the prognosis of lung adenocarcinoma patients. We constructed a prognostic prediction model for lung adenocarcinoma based on IL-1 signalling-related genes using data related to lung adenocarcinoma patients from the GDC, GEO, TISCH2 and TCGA databases, which offers hope for the diagnosis and treatment of lung adenocarcinoma.

## Data availability statement

We sincerely appreciate all members who participated in data collection and analysis. Publicly available datasets were analysed in this study. This data can be found here: https://www.ncbi.nlm.nih.gov/geo/ ,with the accession number GSE58812 and GSE118389.

## Author contributions

YX, NH, and DY designed the study. YX, NH, DY, CY, and WS performed data analysis. YX, NH, and DY drafted and revised the manuscript. All authors contributed to the article and approved the submitted version.
